# Psychiatric signs and symptoms in treatable inborn errors of metabolism

**DOI:** 10.1007/s00415-014-7396-6

**Published:** 2014-08-22

**Authors:** S. Nia

**Affiliations:** Neurological Center Rosenhügel, 2nd Neurological Department, Hospital Hietzing, Karl Landsteiner Institute for Cognitive Neurology and Epilepsy Research, Riedelgasse 5, 1130 Vienna, Austria

**Keywords:** Organic psychosis, Diagnosis, Psychosis, Cognitive impairment, Niemann-Pick disease type C, Metachromatic leukodystrophy

## Abstract

Possible underlying organic causes of psychiatric symptoms can be overlooked in the clinical setting. It is important to increase awareness amongst psychiatric and neurological professionals with regard to certain inborn errors of metabolism as, in some cases, disease-specific therapies are available that can, for instance, treat underlying metabolic causes. The following article describes the basic pathophysiology, clinical and neurological features, and available diagnostic procedures of six treatable metabolic diseases that are associated with neuropsychiatric symptoms: Wilson’s disease, cerebrotendinous xanthomatosis, porphyrias, homocysteinemia, urea cycle disorders, and Niemann-Pick disease type C (NP-C). NP-C is taken as a particularly relevant example because, while it is traditionally considered to be a condition that presents with severe neurological and systemic manifestations in children, an increasing number of patients are being detected who have the adolescent- or adult-onset form, which is frequently associated with neuropsychiatric signs. A notable proportion of adult-onset cases have been reported where NP-C has mistakenly been diagnosed and treated as a psychiatric condition, usually based on patients’ initial presentation with psychotic or schizophrenia-like symptoms. Underlying organic causes of psychiatric disorders such as psychosis should be considered among patients with atypical symptoms and/or resistance to standard therapy. Alongside improved frameworks for additional multidisciplinary diagnostic work in patients with suspected organic disease, the development of convenient and affordable biochemical screening and/or diagnostic methods has enabled new ways to narrow down differential diagnoses.

## Introduction

Inborn errors of metabolism constitute a subgroup of genetic conditions for which disease-specific treatments are increasingly becoming available. Some such conditions present primarily with psychiatric symptoms, but they can be overlooked in a psychiatric setting. In order to prevent diagnostic mistakes an organized framework for screening should be established. In many cases, research into biochemical methods for screening and diagnosis has led to the development of easy and affordable diagnostic possibilities that are not only simple to apply (e.g., based on blood or urine samples) but which also provide quick results. Improved awareness of metabolic diseases within a neuropsychiatric cohort in combination with simple screening tools should easily improve the diagnostic outcome and, therefore, ideally, change the treatment approach.

The following article addresses six treatable inherited metabolic diseases that can initially present with neuropsychiatric symptoms. General pathophysiology, clinical features, and diagnostic methods are summarized with a particular focus on neurological and psychiatric manifestations. Treatment approaches capable of changing disease outcomes and, in some cases, improving prognosis, are covered. Niemann-Pick disease type C (NP-C) is taken as a specific example of an inherited metabolic disease where patients can present initially with isolated psychiatric symptoms over many years before other symptoms occur, and where symptoms can be misdiagnosed as schizophrenia or other psychiatric diseases, based on the widely used international systems for the classification of psychiatric diseases.

In order to enhance diagnostic outcomes, a potential clinical diagnostic algorithm is proposed that could serve as an aid to detecting possible cases of organic psychosis (e.g. as seen in adult-onset NP-C).

## Wilson’s disease

Wilson’s disease (WD) is an autosomal recessive disease that belongs to the group of inherited liver diseases. The incidence in newborns is estimated to be 1:30,000. WD typically includes liver disease that appears in the second decade of life and which is followed by neurological and psychiatric disorders in the third decade [5, 6, 30].

### Genetics and pathogenesis

WD is caused by mutations in the gene encoding ATP7B Cu translocase, which functions as a copper-transporting P-type ATPase that regulates levels of copper in the liver. This enzyme is mainly expressed by hepatocytes and modulates the synthesis of ceruloplasmin. ATP7B Cu translocase is mainly located in the canalicular membrane and helps to excrete copper via bile. In WD, the activity of ATP7B is impaired at the membrane level. Consequently copper accumulates within the hepatocyte. Copper in its free ionic form has a relevant redox potential and induces oxidative stress, attacking cell membranes, proteins, and DNA, particularly in the liver and brain. Generally, the brain is affected symmetrically with excess copper deposition, although symptoms can be worse on one side of the body than another. The copper is often seen most prominently in the basal ganglia [6].

### Clinical features

#### Psychiatric manifestations

Psychiatric features include emotional lability, impulsiveness, disinhibition, and self-injurious behavior. The reported percentage of patients with psychiatric symptoms as the presenting clinical feature is 10–20 %. Psychiatric abnormalities associated with WD have been divided into behavioral, affective, schizophrenia-like, and cognitive. Cognitive impairment is generally mild [1]. Schizophreniform disorders, catatonia, and hallucinations are no more common in WD than in the general population, but psychosis and catatonia occur more commonly in neurological WD [1]. Patients with WD having predominantly neuropsychiatric symptoms, present with symptoms later in life, have a longer time delay from onset of symptoms until definitive diagnosis, and have a poorer outcome compared with patients with hepatic symptoms [8].

#### Neurological manifestations

The most common presenting neurologic feature is asymmetrical tremor, occurring in approximately half of patients. Frequent early symptoms include difficulty speaking, excessive salivation, ataxia, mask-like faces, clumsiness with the hands, and personality changes. Late manifestations include dystonia, spasticity, grand mal seizures, rigidity, and flexion contractures [6].

#### Eye manifestations

A Kayser-Fleischer (K-F) ring is a golden to greenish-brown annular deposition of copper located in the periphery of the cornea. Approximately 95 % of WD patients presenting with neurological signs will have a K-F ring, compared with a relatively smaller proportion (approximately 65 %) of patients who present with hepatic signs [6].

#### Liver manifestations

Liver disease in WD patients is highly variable, ranging from asymptomatic cases with mild hepatomegaly and slight increases in serum hepatocellular enzymes, to chronic liver disease with severe steatosis, up to fibrosis, cirrhosis, and severe liver failure. In approximately two-thirds of cases there is hemolytic anemia, coagulopathy, and/or renal failure [6].

### Diagnosis

The diagnosis of WD is achieved using relatively simple tests that can be applied in symptomatic patients as well as people who show no signs of the disease. These tests include ophthalmologic slit-lamp examination for K-F rings, serum ceruloplasmin test, 24-h urine copper test, liver biopsy for histology and histochemistry and copper quantification, and genetic testing [5, 6, 30]. In symptomatic cases the ‘face of the miniature panda’ is seen within the pontine tegmentum; it is delineated by the relative hypointensity of the medial longitudinal fasciculi and central tegmental tracts in contrast with the hyperintensity of the aqueduct opening into the fourth ventricle bounded inferiorly by the superior medullary velum and superior cerebellar peduncles [36].

### Treatment

With proper therapy, disease progress can be halted and symptoms can be improved [6]. Treatment is aimed at removing excess accumulated copper and preventing its re-accumulation. Chelation therapy drugs approved for treating WD include penicillamine (with maintenance dose 750–1,000 mg/day administered in two divided doses) and trientine (750 or 1,000 mg used for maintenance in two or three divided doses [47]). Both of these drugs act by chelation or binding of copper, causing its increased urinary excretion. Metallothionein inducer drugs are also approved for treating WD. Zinc acts by blocking the absorption of copper in the intestinal tract. This action both depletes accumulated copper and prevents its re-accumulation. Patients with severe hepatitis or liver failure may require a liver transplant [6]. Little is known about treating psychiatric conditions in WD. Clinical improvement in WD with treatment is generally limited to the first 5 years of symptoms [1]. Early recognition and treatment are, therefore, essential.

## Cerebrotendinous xanthomatosis

Cerebrotendinous xanthomatosis (CTX) is a lipid storage disease characterized by infantile-onset diarrhea, childhood-onset cataract, adolescent- to young adult-onset tendon xanthomas, and adult-onset progressive neurological dysfunction and psychiatric disturbances [27, 28].

### Genetics and pathogenesis

CTX is caused by deficiency of the mitochondrial enzyme, sterol 27-hydroxylase, which results in cholestanol and cholesterol accumulation in virtually every tissue. *CYP27A1* is the only gene in which mutations are known to cause CTX. The condition is inherited in an autosomal recessive manner [14, 27, 28].

### Clinical features

#### Neuropsychiatric signs

Intellectual disabilities occur in the early twenties in more than 50 % of individuals. Neuropsychiatric symptoms such as behavioral changes, hallucinations, agitation, aggression, depression, and suicidal attempts may be prominent [27]. Behavioral/personality disorder is the most frequent psychiatric manifestation in all age groups, but especially so in patients younger than 26 years, followed by mood/affective disorder [27]. In patients older than 26 years, psychiatric pathology is more complex, with richer semeiology often in the context of pre-dementia/dementia [13].

#### Neurological signs

Pyramidal signs and/or cerebellar signs become evident at 20–30 years of age [27].

#### Gastrointestinal signs

Chronic diarrhea from infancy may be the earliest clinical manifestation [28].

#### Eye signs

In approximately 75 % of the affected individuals, cataracts are the first finding, usually in the first decade of life [28].

#### Skin signs

Xanthomas associated with the Achilles tendon, the extensor tendons of the elbow and hand, the patellar tendon and the neck tendons appear in the second or third decade of life [28].

### Diagnosis

When clinical signs are evident, CTX is diagnosed by biochemical testing. High plasma and tissue concentrations of cholestanol confirm the diagnosis.

### Treatment

Long-term treatment with chenodeoxycholic acid (CDCA; average dosage 300 mg/day) improves neurophysiological findings [23]. Cataract extraction is usually required. Epilepsy, spasticity, parkinsonism, and psychiatric symptoms are treated symptomatically [28].

## Porphyrias

The porphyrias are inherited metabolic disorders of heme biosynthesis in which specific patterns of heme precursor overproduction are associated with characteristic clinical features. Each type of porphyria is the result of a specific impairment of the activity of one of the enzymes involved in heme biosynthesis. Porphyrias are classified as erythropoietic or hepatic in type, depending on the primary organ in which excess production of porphyrins or their precursors takes place [16].

### Genetics and pathogenesis

The porphyrias are inherited by a dominant autosomal mechanism, except in the cases of congenital erythropoietic porphyria (CEP) and delta-aminolevulinic acid dehydratase (ALAD) deficiency, which are inherited by a recessive autosomal mechanism. Not all gene carriers of inherited porphyrias develop clinical disease, and there is a significant interplay between the primary gene defect and the secondary acquired or environmental factors (e.g., medication, liver damage, hormonal changes and starvation) [16].

Heme pathway intermediates are potentially toxic. Their overproduction causes the characteristic neurovisceral and/or photosensitizing symptoms. Porphyrins produce free radicals when exposed to ultraviolet light. As a result, skin damage ensues in light-exposed areas resulting in cutaneous porphyrias. In contrast to porphyrins, their precursors are associated with neurological symptoms or acute hepatic porphyrias. Porphyrins and their precursors are excreted in urine or stool depending on their solubility. Accordingly, the water-soluble uroporphyrin is excreted in urine, while the water-insoluble protoporphyrin is excreted via bile and stool. Coproporphyrin is excreted into both urine and stool because of its intermediate solubility. Porphyrin precursors are essentially all excreted in urine. During an acute attack of porphyria an increased production of heme precursors leads to the accumulation of porphyrin intermediates [7, 16].

### Clinical features

#### Erythropoietic porphyrias

Porphyrins in red blood cells can cause photosensitive cell lysis, resulting in hemolytic anemia. The two homozygous erythropoietic porphyrias, CEP and hepato-erythropoietic porphyria (HEP), are associated with hemolytic anemia of varying degrees. In contrast, erythropoietic protoporphyria (EPP), a heterozygous disease, rarely has accompanying hemolytic anemia. The effect of life-long anemia in CEP or HEP may lead to compensatory expansion of erythroid marrow, which may result in pathological fractures, vertebral compression or collapse, and shortness of stature. The hemolysis is also associated with varying degrees of splenomegaly and the production of pigment-laden gallstones.

#### Acute hepatic porphyrias

The most common neurovisceral complaints in acute hepatic porphyrias are abdominal pain, vomiting, constipation, muscle weakness, mental symptoms, head/neck/chest pain, hypertension, tachycardia, convulsions, sensory loss, fever, respiratory paralysis, and diarrhea [16].

#### Chronic hepatic porphyria

Patients with chronic hepatic porphyria, such as Porphyria cutanea tarda (PCT), are associated with significant chronic cutaneous photosensitivity but they do not accompany neurological symptoms. Petechiae and purpuric lesions may occur. Skin lichenification, leathery pseudovesicles, and nail changes can be pronounced. Chronic blistering lesions may develop; the fluid-filled vesicles rupture easily and the denuded areas become crusted and heal slowly; secondary infection is common. Previous areas of blisters may appear atrophic, or brownish. Facial hypertrichosis, scarring, and hyperpigmentation can also occur [7, 38].

#### Psychiatric symptoms

Psychiatric symptoms in porphyria have led to controversial discussion [16]. In the past, reported psychiatric manifestations have included hysteria, anxiety, depression, phobias, psychosis, organic disorders, agitation, delirium, and altered consciousness ranging from somnolence to coma. Some patients were described with psychosis similar to schizophrenia [3]. Psychiatric manifestations may be divided into two groups. First, an association between chronic porphyria and mild anxiety and depression has been postulated [4]; the nature of this association is yet to be explained. Second, during the active phase of the acute attack, clinically obvious psychiatric symptoms such as psychosis, anxiety, depression, agitation, and delirium are common and may be present in up to 30 % of patients [10, 15, 34]. The cause of these manifestations is almost certainly multifactorial and may include common effects of medication used in the management of the acute attack, such as sedation and disorientation arising from the use of opioids, and the effects of metabolic disturbances associated with the attack (e.g., hypernatremia). Acute psychotic manifestations such as paranoia and hallucinations can occur but are very uncommon [16].

### Diagnosis

The diagnosis of an acute attack of porphyria requires the demonstration of increased urinary excretion of the heme precursor, porphobilinogen (PBG). Molecular diagnostic testing is powerful and useful in kindred evaluation and genetic counseling when a disease-causing mutation has been identified in the family. It is also the only proper way to screen asymptomatic gene carriers [9, 40].

### Treatment

Recognition and avoidance of precipitating events is a key initial part of treatment. Acute attacks of hepatic porphyrias should be treated by providing sufficient amounts of carbohydrate-derived calories (e.g., by glucose infusion), and intravenous infusion of hematin [34]. Hemolytic anemia in erythropoietic porphyrias may be treated by blood transfusion. Cutaneous photosensitivity of erythropoietic protoporphyria may be treated using oral β-carotene, while photosensitivity associated with PCT can be treated by phlebotomy or oral chloroquine. Liver transplantation can be temporarily beneficial, but transplanted livers are susceptible to protoporphyrin-induced damage [9, 16, 40]. Avoiding precipitating factors such as the use of illicit drugs, excessive alcohol consumption, smoking, and severe calorie restriction can help to prevent attacks. Additionally the maintenance of a regular, balanced diet, prompt treatment of infections, and reduction of stress can further reduce attacks [49].

## Homocysteinemia

High levels of homocysteine are associated with cerebrovascular disease, decreased levels of monoamine neurotransmitters, and depression of mood.

### Genetics and pathogenesis

Homocysteine is a sulfurated amino acid derived from ingested methionine found in cheeses, eggs, fish, meat, and poultry. It is directly toxic to neurons and blood vessels and can induce DNA strand breakage, oxidative stress, and apoptosis. The pathway produces methyl groups required for the synthesis of catecholamines and DNA. This is accomplished by remethylating homocysteine—using vitamin B_12_ and folate as cofactors—back to methionine. Homocysteine is cleared by transulfuration to cysteine and glutathione, an important antioxidant. Transulfuration requires vitamins B_6_ and B_12_.

The components of the homocysteine-methionine cycle, as well as cysteine and glutathione and the enzymes of the pathway, are affected by genetic variation, diet, kidney and gastrointestinal diseases, and prescribed and over-the-counter drugs. Since homocysteine is a sensitive indicator of B vitamin deficiency, an elevated homocysteine level is a marker for a pathogenic process as well as for a cause of pathology. Serum levels of homocysteine increase after methionine loading; in dietary deficiency of vitamin B_12_, folate, and vitamin B_6_ and because of renal disease and genetic variation of the enzymes essential for the metabolism of homocysteine (e.g., methyl-tetrahydro-folate reductase [*MTHFR*] and cystathionine beta-synthase [*CBS*]). Other causes of hyperhomocysteinemia include gastric atrophy, inflammatory bowel disease, and laxative use.

Severe hyperhomocysteinemia predisposes patients to complications of arterial and venous vascular disease, with approximately 50 % of patients developing stroke, myocardial infarction, or pulmonary embolism before 30 years of age [32]. The age at onset and severity of these clinical features vary widely among affected individuals.

### Clinical features

#### Neuropsychiatric abnormalities

Moderate hyperhomocysteinemia has also been associated with an increased risk of the development of dementia. An association with vascular dementia seems obvious. However, some studies have suggested that Alzheimer disease, in particular, is associated with hyperhomocysteinemia. There is evidence for the association between homocysteine levels and depression, vascular disease, and neurotransmitters [35].

#### Vascular abnormalities

Moderate hyperhomocysteinemia is a risk factor for stroke, peripheral vascular disease, myocardial infarction, and venous thromboembolism [32].

#### Ocular abnormalities

Mild hyperhomocysteinemia is established as an independent risk factor for atherothrombotic disease, including ocular pathologies such as retinal vascular occlusion and non-arteritic ischemic optic neuropathy (54).

#### Skeletal abnormalities

Hyperhomocysteinemia can impair the parasympathetic and sympathetic regulation in the blood vessels of skeletal muscle and affect long-term muscle function [46].

### Diagnosis

A neonatal screening test, called the Guthrie test, detects high levels of methionine in heel-stick blood. Quantitative tests for homocysteine in urine and blood are also available. Measurement of CBS activity in cultured fibroblasts provides definitive support for the diagnosis. The risk for vascular disease is graded with respect to the level of homocysteine; however, there is no generally accepted abnormal threshold value. If homocysteinemia is determined by a test, then tests for deficiency in folic acid, vitamin B_12_, and pyridoxine may also be performed. Genetic abnormalities are reported on chromosome 1 pertaining to MTHFR. However, the mere presence of this abnormality may not confer a risk for vascular disease [12, 35].

### Treatment

Hyperhomocysteinemia can be treated directly with vitamin supplementation. The primary vitamin used to lower homocysteine levels is folate. A trial of homocysteine therapy and stroke risk, which randomized 5,522 adults with known cardiovascular disease to a daily treatment regimen of B-vitamin therapy (2.5 mg of folic acid, 50 mg of vitamin B_6_, and 1 mg of vitamin B_12_) for 5 years, achieved a 25 % reduction in the risk of stroke [39].

Enzyme-inducers of cytochrome P450 like carbamazepine or phenytoin should be avoided since they lead to an increased homocystein level. In cases where antiepileptics are needed, switching to non-inducing drugs like levetiracetam or lamotrigine can be helpful [26].

## Urea cycle disorders

Urea cycle disorders (UCD) result from defects in the metabolism of waste nitrogen from the breakdown of protein and other nitrogen-containing molecules. The severity of the urea cycle defect is influenced by the position of the defective enzyme in the nitrogen metabolic pathway and the severity of the enzyme defect [24]. Hereditary UCD may present at any age, with a prevalence of 1:10,000. In addition to forms that present in the neonatal period and in young children, there are forms that manifest later, during adolescence or early adulthood.

### Genetics and pathophysiology

Deficiencies are mainly inherited in an autosomal recessive manner. Severe deficiency results in the accumulation of ammonia and other precursor metabolites during the first few days of life.

### Clinical features

#### Psychiatric manifestations

These diseases may be associated with confusion, behavioral disorders or hallucinations suggestive of an atypical form of depression [2, 36], an acute psychotic disorder, or even resembling schizophrenia [11]. The psychiatric symptoms that occur are nearly always accompanied by headache and/or gastrointestinal symptoms (nausea, vomiting). UCD may present initially with postpartum psychiatric symptoms and may represent an under-recognized cause of postpartum psychosis [11].

#### Other clinical symptoms

Infants with a severe UCD are normal at birth but rapidly develop cerebral edema and the related signs of lethargy, anorexia, hyper- or hypo-ventilation, hypothermia, seizures, neurologic posturing, and coma. In milder (or partial) deficiencies ammonia accumulation may be triggered by illness or stress at almost any time of life. In these disorders the elevations of plasma ammonia concentration and symptoms are often subtle and the first recognized clinical episode may not occur for months or decades [24]. Patients often have protein intolerance and those with later-onset disease often spontaneously change their diet, becoming vegetarians or anorexic [36].

### Diagnosis

The diagnosis of a UCD is based on clinical suspicion as well as biochemical and molecular genetic testing. Carrier testing for at-risk relatives and prenatal testing for pregnancies at increased risk using molecular genetic testing are possible for any of the UCDs if the disease-causing mutation(s) in the family are known. A plasma ammonia concentration of 150 μmol/L or higher, associated with a normal anion gap and a normal plasma glucose concentration, is an indication for the presence of a UCD. Molecular genetic testing is possible for all UCDs [24].

### Treatment

In acute severe hyperammonemia, dialysis and hemofiltration are necessary to reduce plasma ammonia concentration. Additionally, intravenous administration of arginine hydrochloride and nitrogen scavenger drugs allows alternative pathway excretion of excess nitrogen. Other treatment strategies include restriction of protein for 12–24 h to reduce dietary nitrogen intake; supply of dietary calories as carbohydrates and fat, and physiological stabilization with intravenous fluids and cardiac pressors, while avoiding over-hydration.

Long-term management involves the prevention of catabolism to avoid hyperammonemic episodes using dietary protein restriction, specialized formulas, and oral nitrogen-scavenging drugs. In order to prevent secondary complications, it is useful to minimize the risk of respiratory and gastrointestinal illnesses by routine immunizations; multivitamin and fluoride supplementation, and the appropriate use of antipyretics. Agents such as valproic acid, prolonged fasting or starvation, intravenous steroids, and large boluses of protein or amino acids should be avoided [24].

## Niemann Pick disease type C

Niemann Pick disease type C (NP-C) serves as a good example of a treatable metabolic disease with psychiatric symptoms, and with a representative diagnostic work up for the re-evaluation of initial psychiatric diagnoses.

### Genetics and pathogenesis

The underlying genetic and biochemical causes of this disease have been revised in detail elsewhere [45] and in this supplement (see [25]).

### Clinical features

#### Psychiatric signs

Special attention should be paid with regard to NP-C in patients with adolescent- or adult-onset of neurological symptoms. In comparison to patients with childhood onset, disease progression among patients with later-onset disease is less aggressive [31, 52]. Psychiatric symptoms may dominate the initial clinical impression. Decades can pass before any neurological manifestations become obvious and lead to a reconsideration of possible initial psychiatric diagnosis [44].

In the psychiatric setting, the awareness of organic causes of psychiatric symptoms may be lacking, particularly with regard to possible associated rare diseases. Even though physical examination and brain imaging are part of the diagnostic work up, a necessary re-evaluation of patients’ status may not be repeated often enough over time. The clinical picture may change and neurological deficits that were absent at first presentation may emerge as the disease progresses [18]. In addition, some movement disorders and cognitive deficits may be misinterpreted as part of the possible side effects of long-term medications used in psychiatric cases [20]. Also the differentiation of cognitive decline between, for instance, a depressed or psychotic patient and a patient with an organic disease can be challenging. Psychological test batteries can provide some details regarding the nature of cognitive or psychiatric deficits, but distinct proof is often still missing [21]. Hence there is a significant need for easily accessible screening methods for adolescent and adult-onset NP-C. In addition, follow-up physical examinations and brain imaging studies are essential.

Psychiatric symptoms associated with NP-C vary widely [18, 31, 42, 45]. Case studies of adolescent/adult-onset NP-C have reported the occurrence of psychiatric symptoms decades before the patient has been admitted to a neurology department [18, 20, 44]. Patients with adolescent-onset disease primarily show learning disabilities and/or behavioral problems [17, 31, 45]. Among adult-onset patients affective disorders, psychosis and cognitive decline are most often recorded and result in social isolation and performance decline at work and in daily routine activities [18, 31, 45].

Patients exhibiting psychosis can show a visual hallucinatory phenotype, although other hallucinatory forms have been described [47]. In one case initial presentation of the disorder was at the age of 21, with cognitive decline, perseverations, thought disorders, and incongruity of affect. At that time the patient was diagnosed with hebephrenic schizophrenia. Six years later he presented with supranuclear vertical gaze palsy, saccadic eye movements, bradykinesia, dysarthria, and ataxia, which finally led to the diagnosis of NP-C [29]. In another case report an 8-year-old NP-C patient was described with auditory hallucinations and 7 years later developed a typical paranoid schizophrenic illness that was only partially responsive to risperidone [37].

Treatment resistance to psychiatric medication (e.g., with antipsychotics) is another indicator of possible organic psychosis related to NP-C. Difficulty in classifying presenting psychiatric symptoms to a known psychiatric disease can provide a further clue as to a possible organic cause for psychosis. In such cases, the necessary diagnostic criteria are often not fulfilled, leading to confusion and arrival at a number of different diagnoses over time.

#### Neuropsychological findings

Cognitive decline features in almost all cases of adolescent and adult-onset NP-C and generally fulfills criteria for dementia as the disease progresses, often starting with difficulties in word fluidity and executive dysfunction [31, 41, 45]. Sleep disturbance can occur [48].

### Diagnosis and disease management: a psychiatric perspective

Processes for screening, diagnosis, and treatment of NP-C are covered in detail elsewhere [31, 45, 51] and in this supplement (see [25] [43]). However, from a psychiatric perspective, no general psychopathological profile has been proposed to date that describes neuropsychiatric symptoms in NP-C. However, based on reported findings, a patient with cognitive dysfunction, missing clear psychiatric diagnostic criteria, and therapy resistance should be re-evaluated and diagnostic work up should be repeated to rule out NP-C as a possible cause. A second, specialist opinion can be helpful in this respect. Further, brain imaging can reveal general brain atrophy, although this may only be seen in the later stages of NP-C. Data indicate that, in cases where there is a high suspicion of NP-C, plasma oxysterol testing can also be useful [19, 33].

Special attention should be paid in cases where there is a suspected diagnosis of ‘hebephrenic schizophrenia’ (also called ‘disorganized schizophrenia’). The suggested diagnostic criteria for this form of schizophrenia in the World Health Organization (WHO) ICD-10 diagnostic criteria include changes in affect, drive, and thought disorders [50]. All of the described symptoms may occur in patients with NP-C or another metabolic disease with psychiatric symptoms, as described in the sections above (e.g., metachromatic leukodystrophy [22]). The diagnosis of disorganized schizophrenia itself should lead to a repeated diagnostic work up, particularly in view of renewed recent discussions regarding the existence of hebephrenic schizophrenia.

## Conclusion

**Fig. 1:**
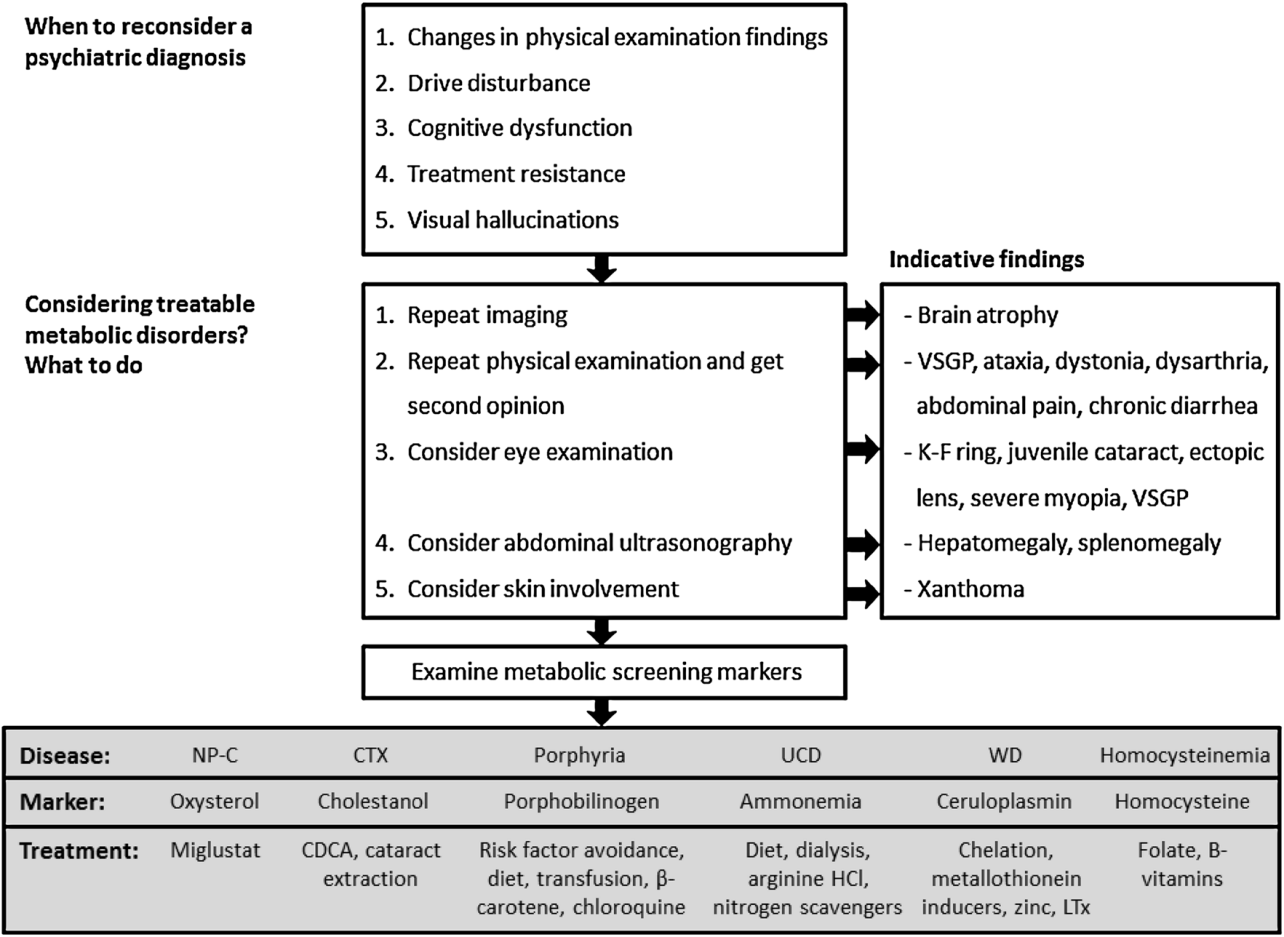
Proposed algorithm defining indicative diagnostic tests for patients with possible treatable causes of organic psychiatric disease. *CTX* Cerebrotendinous xanthomatosis; *K-F* Kayser-Fleischer (ring); *LTx* liver transplantation; *NP-C* Niemann-Pick disease type C, *UCD* urea cycle disorders, *VSGP* vertical supranuclear gaze palsy, *WD* Wilson’s disease

A number of inherited metabolic diseases have been described that can initially present with psychiatric signs and symptoms. Intensive research efforts over the past three decades have succeeded in defining disease-specific treatment strategies in a limited proportion of these diseases. Increased awareness of possible underlying causes of organic psychiatric disorders is required to expedite revised diagnoses and to allow the appropriate application of these available therapies. A proposed algorithm for patients with a number of relevant treatable inherited metabolic conditions is included in Fig. 1. In particular, organic causes of psychosis should be considered among patients with atypical psychiatric symptoms and/or resistance to standard therapy. After further diagnostic processes, easy-to-apply screening tests are now available that can assist in confirming diagnoses.

## References

[1] Akil M, Brewer GJ (1995). Psychiatric and behavioral abnormalities in Wilson’s disease. Adv Neurology.

[2] Arn PH, Hauser ER, Thomas GH, Herman G, Hess D, Brusilow SW (1990). Hyperammonemia in women with a mutation at the ornithine carbamoyltransferase locus. A cause of postpartum coma. N Engl J Med.

[3] Burgovne K, Swartz R, Ananth J (1995). Porphyria: reexamination of psychiatric implications. Psychotherapy Psychosom.

[4] Bylesjo I, Wikberg A, Andersson C (2009). Clinical aspects of acute intermittent porphyria in northern Sweden: a population-based study. Scand J Clin Lab Invest.

[5] Cox DW (1999). Disorders of copper transport. Br Med Bull.

[6] Cox DW (2010). Wilson disease.

[7] Crimlisk HL (1997). The little imitator–porphyria: a neuropsychiatric disorder. J Neurol Neurosurg Psychiatry.

[8] Dening TR, Berrios GE (1989). Wilson’s disease. Psychiatric symptoms in 195 cases. Arch Gen Psychiatry.

[9] Deybach JC, Badminton M, Puy H, Sandberg S, Frank J, Harper P, Martasek P, Minder E, Parker S, Thunell S, Elder G (2006). European porphyria initiative (EPI): a platform to develop a common approach to the management of porphyrias and to promote research in the field. Physiol Res.

[10] Elder GH, Hift RJ, Meissner PN (1997). The acute porphyrias. Lancet.

[11] Enns GM, O’Brien WE, Kobayashi K, Shinzawa H, Pellegrino JE (2005). Postpartum “psychosis” in mild argininosuccinate synthetase deficiency. Obstet Gynecol.

[12] Folstein M, Liu T, Peter I, Buell J, Arsenault L, Scott T, Qiu WW (2007). The homocysteine hypothesis of depression. Am J Psychiatry.

[13] Fraidakis MJ (2013). Psychiatric manifestations in cerebrotendinous xanthomatosis. Transl Psychiatry.

[14] Gallus GN, Dotti MT, Federico A (2006). Clinical and molecular diagnosis of cerebrotendinous xanthomatosis with a review of the mutations in the CYP27A1 gene. Neurol Sci.

[15] Gonzalez-Arriaza HL, Bostwick JM (2003). Acute porphyrias: a case report and review. Am J Psychiatry.

[16] Hift RJ, Peters TJ, Meissner PN (2012). A review of the clinical presentation, natural history and inheritance of variegate porphyria: its implausibility as the source of the ‘Royal Malady’. J Clin Pathol.

[17] Imrie J, Dasgupta S, Besley GT, Harris C, Heptinstall L, Knight S, Vanier MT, Fensom AH, Ward C, Jacklin E, Whitehouse C, Wraith JE (2007). The natural history of Niemann-Pick disease type C in the UK. J Inherit Metab Dis.

[18] Imrie J, Vijayaraghaven S, Whitehouse C, Harris S, Heptinstall L, Church H, Cooper A, Besley GT, Wraith JE (2002). Niemann-Pick disease type C in adults. J Inherit Metab Dis.

[19] Jiang X, Sidhu R, Porter FD, Yanjanin NM, Speak AO, te Vruchte DT, Platt FM, Fujiwara H, Scherrer DE, Zhang J, Dietzen DJ, Schaffer JE, Ory DS (2011). A sensitive and specific LC-MS/MS method for rapid diagnosis of Niemann-Pick C1 disease from human plasma. J Lipid Res.

[20] Josephs KA, Van Gerpen MW, Van Gerpen JA (2003). Adult onset Niemann-Pick disease type C presenting with psychosis. J Neurol Neurosurg Psychiatry.

[21] Klarner B, Klunemann HH, Lurding R, Aslanidis C, Rupprecht R (2007). Neuropsychological profile of adult patients with Niemann-Pick C1 (NPC1) mutations. J Inherit Metab Dis.

[22] Kumperscak HG, Paschke E, Gradisnik P, Vidmar J, Bradac SU (2005). Adult metachromatic leukodystrophy: disorganized schizophrenia-like symptoms and postpartum depression in 2 sisters. J Psychiatry Neurosci.

[23] Kuriyama M, Tokimura Y, Fujiyama J, Utatsu Y, Osame M (1994). Treatment of cerebrotendinous xanthomatosis: effects of chenodeoxycholic acid, pravastatin, and combined use. J Neurol Sci.

[24] Lanpher BC, Gropman A, Chapman KA, Lichter-Konecki U, Urea Cycle Disorders Consortium, Summar ML (1993) In: Urea cycle disorders overview, Pagon RA, Adam MP, Ardinger HH, Bird TD, Dolan CR, Fong CT, Smith RJH, Stephens K (eds). GeneReviews^®^, Seattle (WA) 20301396

[25] McKay K, Gissen P (2014) Genetic and laboratory diagnostic approach in Niemann Pick disease type C. J Neurol. doi:10.1007/s00415-014-7386-8 (this issue) 10.1007/s00415-014-7386-8 PMC4141153 25145893

[26] Mintzer S, Skidmore CT, Abidin CJ, Morales MC, Chervoneva I, Capuzzi DM, Sperling MR (2009). Effects of antiepileptic drugs on lipids, homocysteine, and C-reactive protein. Ann Neurol.

[27] Moghadasian MH (2004). Cerebrotendinous xanthomatosis: clinical course, genotypes and metabolic backgrounds. Clin Invest Med.

[28] Moghadasian MH, Salen G, Frohlich JJ, Scudamore CH (2002). Cerebrotendinous xanthomatosis: a rare disease with diverse manifestations. Arch Neurol.

[29] Nia S, Geiblinger S, Gruber C, Gallmetzer P, Gerschlager W, Baumgartner C (2011). Psychiatric symptoms as an early sign of the adult form of lysosomal storage disease Niemann-Pick type C. J Neurol Neurochirurg Psych.

[30] NIDDK (2009) National Institute for Diabetes and Digestive and Kidney Diseases. Wilson disease. http://digestive.niddk.nih.gov/ddiseases/pubs/wilson/. Accessed 28 May 2014

[31] Patterson MC, Hendriksz CJ, Walterfang M, Sedel F, Vanier MT, Wijburg F, Group N-CGW (2012). Recommendations for the diagnosis and management of Niemann-Pick disease type C: an update. Mol Genet Metab.

[32] Petras M, Tatarkova Z, Kovalska M, Mokra D, Dobrota D, Lehotsky J, Drgova A (2014). Hyperhomocysteinemia as a risk factor for the neuronal system disorders. J Physiol Pharmacol (Off J Pol Physiol Soc).

[33] Porter FD, DE Scherrer, Lanier MH, Langmade SJ, Molugu V, Gale SE, Olzeski D, Sidhu R, Dietzen DJ, Fu R, Wassif CA, Yanjanin NM, Marso SP, House J, Vite C, Schaffer JE, Ory DS (2010). Cholesterol oxidation products are sensitive and specific blood-based biomarkers for Niemann-Pick C1 disease. Sci Transl Med.

[34] Puy H, Gouya L, Deybach JC (2010). Porphyrias. Lancet.

[35] Reif A, Schneider MF, Kamolz S, Pfuhlmann B (2003). Homocysteinemia in psychiatric disorders: association with dementia and depression, but not schizophrenia in female patients. J Neural Trans.

[36] Roberts EA, Schilsky ML (2008). Diagnosis and treatment of Wilson disease: an update. Hepatology.

[37] Sandu S, Jackowski-Dohrmann S, Ladner A, Haberhausen M, Bachmann C (2009). Niemann-Pick disease type C1 presenting with psychosis in an adolescent male. Eur Child Adolesc Psychiatry.

[38] Santosh PJ, Malhotra S (1994). Varied psychiatric manifestations of acute intermittent porphyria. Biol Psychiatry.

[39] Saposnik G, Ray JG, Sheridan P, McQueen M, Lonn E (2009). Heart outcomes prevention evaluation 2 investigators homocysteine-lowering therapy and stroke risk, severity, and disability: additional findings from the HOPE 2 trial. Stroke.

[40] Sassa S (2006). Modern diagnosis and management of the porphyrias. Br J Haematol.

[41] Sedel F, Baumann N, Turpin JC, Lyon-Caen O, Saudubray JM, Cohen D (2007). Psychiatric manifestations revealing inborn errors of metabolism in adolescents and adults. J Inherit Metab Dis.

[42] Sevin M, Lesca G, Baumann N, Millat G, Lyon-Caen O, Vanier MT, Sedel F (2007). The adult form of Niemann-Pick disease type C. Brain.

[43] Strupp M, Kremmyda O, Adamczyk C, Böttcher N, Muth C, Yip CW, Bremova T (2014) Central ocular motor disorders, including gaze palsy and nystagmus. J Neurol. doi:10.1007/s00415-014-7385-9 (this issue) 10.1007/s00415-014-7385-9 PMC4141156 25145891

[44] Trendelenburg G, Vanier MT, Maza S, Millat G, Bohner G, Munz DL, Zschenderlein R (2006). Niemann-Pick type C disease in a 68-year-old patient. J Neurol Neurosurg Psychiatry.

[45] Vanier MT (2010). Niemann-Pick disease type C. Orphanet J Rare Dis.

[46] Veeranki S, Tyagi SC (2013). Defective homocysteine metabolism: potential implications for skeletal muscle malfunction. Int J Mol Sci.

[47] Walterfang M, Fietz M, Abel L, Bowman E, Mocellin R, Velakoulis D (2009). Gender dimorphism in siblings with schizophrenia-like psychosis due to Niemann-Pick disease type C. J Inherit Metab Dis.

[48] Walterfang M, Fietz M, Fahey M, Sullivan D, Leane P, Lubman DI, Velakoulis D (2006). The neuropsychiatry of Niemann-Pick type C disease in adulthood. J Neuropsychiatry Clin Neurosci.

[49] Whatley SD, Badminton MN (2014) Acute intermitted porphyria. GeneReviews^®^. http://www.ncbi.nlm.nih.gov/books/NBK1193/. Accessed 28 May 2014

[50] WHO (2010) International Statistical Classification of Diseases and Related Health Problems, 10th Revision. http://apps.who.int/classifications/icd10/browse/2010/en. Accessed 28 May 2014

[51] Wijburg FA, Sedel F, Pineda M, Hendriksz C, Fahey M, Watlerfang M, Patterson MC, Wraith JE (2011). Suspicion index to aid diagnosis of Niemann-Pick type C disease (NP-C), as autosomal recessive neurovisceral disorder. J Inherit Metab Dis.

[52] Wraith JE, Guffon N, Rohrbach M, Hwu WL, Korenke GC, Bembi B, Luzy C, Giorgino R, Sedel F (2009). Natural history of Niemann-Pick disease type C in a multicentre observational retrospective cohort study. Mol Genet Metab.

